# Surprising visit: the rare plasma cell variant

**DOI:** 10.1097/BS9.0000000000000218

**Published:** 2025-02-11

**Authors:** Snehil Kumar, Faiq Ahmed, Manasi Mundada, Suseela Kondandapani, Pavan Kumar

**Affiliations:** a Department of Pathology, Basavatarakam Indo-American Cancer Hospital and Research Institute, Hyderabad, Telangana, India; b Department of Medical Oncology, Basavatarakam Indo-American Cancer Hospital and Research Institute, Hyderabad, Telangana, India

## 1. INTRODUCTION

Multiple myeloma is a neoplastic plasma cell dyscrasia characterized by anemia, monoclonal protein in the serum or urine, bone lesions, hypercalcemia, and renal insufficiency.^1^ A bone marrow aspirate or biopsy showing clonal plasma cells is one of the key components for the diagnosis of myeloma. The morphologic appearance of neoplastic plasma cells can be often indistinguishable from normal plasma cells under a light microscopy. However, loss of perinuclear hof, presence of various forms like binucleate or multinucleate cells, inclusion owing to cytoplasmic immunoglobulin can provide subtle diagnostic clues. Rarely they can also present with unusual forms like anaplastic variant, with auer rod-like inclusion, small-cell variant, or having intracytoplasmic coarse azurophilic granules leading to erroneous assessment, potentially delaying accurate diagnosis.^2^ We report a case having plasma cell variant with very coarse azurophilic granules masquerading the diagnosis.

## 2. CASE REPORT

A 49-year-old lady with a history of persistent fever, myalgia, and bilateral lower limb pain on evaluation was found to have anemia, leukocytosis, and thrombocytopenia. She was being treated as suspicion of hematological malignancy with no response and was referred to our hospital for further management. At presentation, she had paraparesis with severe back pain and clinical features of acute kidney injury. Magnetic resonance imaging (MRI) spine revealed a posterior disc bulge of C5 to C6 intervertebral disc, effacing the anterior thecal sac and compressing the spinal cord diffuse along with altered marrow signal intensity in the cervical, dorsal, lumbar, and sacral vertebrae having a suspicion of a leukemic infiltration. Multiple lytic lesions in axial and appendicular skeleton were also seen. Computed tomography scan (CT scan) abdomen showed a bulky edematous kidney.

Initial diagnostic investigation revealed severe anemia with a hemoglobin of 5.8 g/dL and a marked thrombocytopenia with a platelet count of 34,000/cumm. Peripheral examination showed marked rouleaux formation and 54% atypical cells showing high nuclear to cytoplasmic ratio with multiple azurophilic granules in the cytoplasm. Other blood investigation showed increased total protein with an altered albumin-to-globulin ratio and high serum creatinine and calcium levels (Table 1). All the investigations strongly pointed toward a diagnosis of plasma cell myeloma and further myeloma markers including serum electrophoresis was done. Serum lactate dehydrogenase was slightly raised 260 U/L (120–246) with a very high β2 microglobulin >40 mg/L (0.81–2.19). The serum protein electrophoresis revealed a monoclonal band with M-protein of 6.1 g/dL, a serum and urine light chain free assay showing κ to λ ratio of 741.07 (0.26–1.65) and 635.58 (2.04–10.37), respectively.

**Table 1:** Serum test results of the patient at initial presentation.

Investigation	Patient result	Normal range
Serum total protein	11.5	5.70–8.00 g/dL
Serum albumin	3.3	3.50–5.00 g/dL
Serum globulin	8.2	1.90–3.70 g/dL
Serum creatinine	3.86	0.52–1.04 mg/dL
Serum calcium	13.2	8.40–10.20 mg/dL

**Figure 1.**
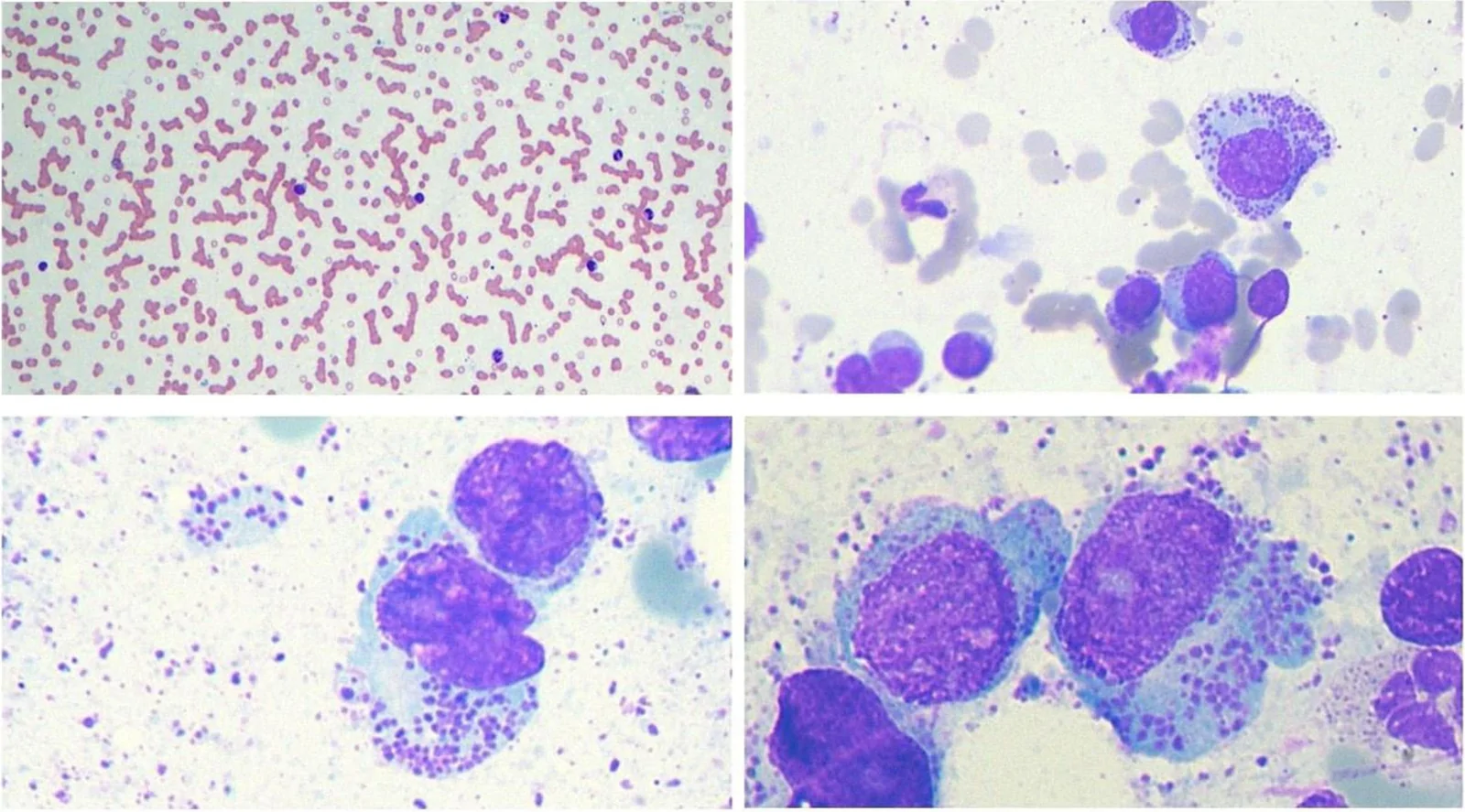
Peripheral smear showing marked rouleaux formation. Bone marrow aspirate showing neoplastic plasma cell with numerous azurophilic granules in cytoplasm.

**Figure 2.**
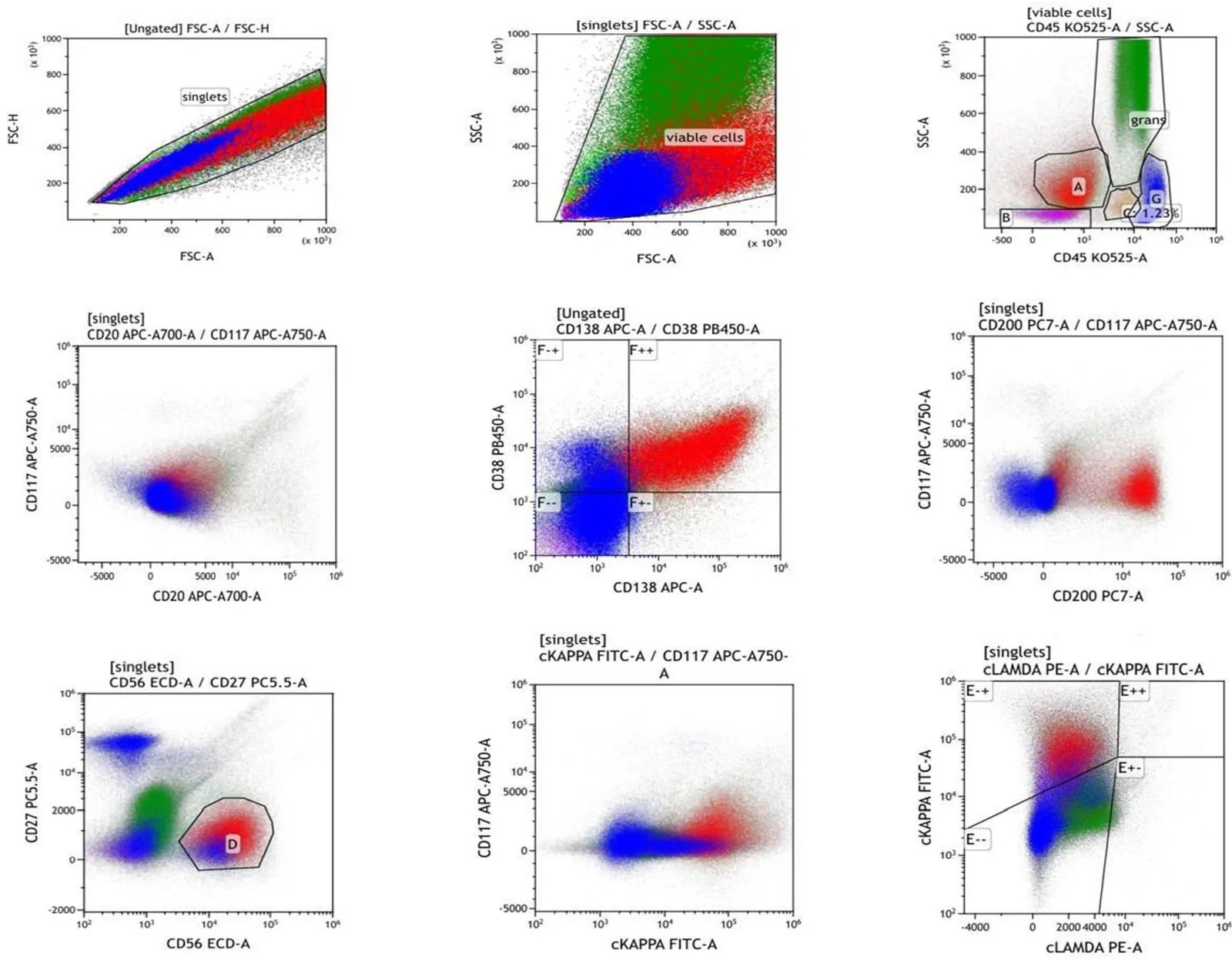
Flow cytometry plots showing κ restriction of plasma cells.

The patient further underwent a bone marrow examination, flow cytometry, and fluorescence in situ hybridization (FISH) study. The bone marrow aspirate and biopsy showed hypercellularity for age composed of reduced trilineage maturing hematopoiesis with replacement of marrow by sheets of plasma cells showing eccentrically placed nucleus with numerous ovoid-shaped intracytoplasmic coarse azurophilic granules mimicking mast cells (**Fig. 1**). These granules stained negative for special stains like myeloperoxidase and toluidine blue. Flow cytometry done on the peripheral blood and bone marrow aspirate showed neoplastic plasma cells with the following phenotype CD45-, CD19-, CD117-, CD20 dim, CD27 variable expression, CD38 positive, CD56 positive, CD138 positive, CD200 positive, and Ig kappa restriction (**Fig. 2**). Immunohistochemistry performed on bone marrow biopsy showed strong positivity for CD138 and faint positivity for MUM1 also revealing a plasma cell phenotype. Bone marrow aspirate was sent for FISH characterization which revealed evidence of *IGH::FGFR3*-t(4;14)(p16;q32) with a frequency of 87%. Other FISH abnormalities observed were hyperploidy with 3 to 5 copies of chromosome 3 (frequency of 85%), 4 to 5 copies of chromosome 5 (frequency of 86%), tri-tetrasomy of chromosome 7 (frequency of 80%), 4 to 6 copies of chromosome 9 (frequency of 86%), 4 copies each of CCND1 alleles and chromosome 11 (frequency of 86%), 3 to 6 copies of chromosome 15 (frequency of 86%), and 3 copies each of MAF alleles and chromosome 16 (frequency of 86%).

## 3. DISCUSSION

Neoplastic plasma cells found in multiple myeloma are characterized by extremely high diversity of morphology.^3^ There are numerous identities that plasma cells can assume. Certain plasma cells bear resemblance to hematological neoplasms, such as B- and T-cell lymphomas, acute leukemia, and myeloproliferative neoplasms, while others are similar to non-hematological neoplasms, including melanoma, carcinoma, and sarcoma. It is important for pathologists to be aware of various morphological variants of the plasma cells to avoid an inaccurate diagnosis. Challenging morphological variants of plasma cells requires to be confirmed with flow cytometric immunophenotyping or immunohistochemistry. Literature has described various cytological variations such as polymorphous, blastic, small cells, anaplastic, signet-ring cells, histiocytoid cells, clear cells, and spindle cells.^4^ In our case, we describe a rare variant with azurophilic granules previously described in few case reports named as Snapper Schneid granules. Such inclusion bodies were first described by Snapper et al^5^ in 1947.^6^ He described that the cytoplasm of myeloma cells is rich in ribose nucleic acid and these inclusion bodies were developed in myeloma cells under the influence of stilbamidine. On the contrary, several other authors believe these granules as precipitated immunoglobulins or lysosomal in origin.^7^ Such azurophilic granules were also described in reactive plasmacytosis suggesting these as deposition of excess immunoglobulin. In contrast, azurophilic granules of neutrophils contain a variety of enzymes including myeloperoxidase and neutrophil elastase that are active both extracellularly and intracellularly. Table 2 summarizes the clinical characteristics of previously reported multiple myeloma patients with azurophilic granules. With the above morphology described, we had a differential diagnosis of mast cell leukemia, aggressive natural killer (NK)/T-cell lymphoma, and abnormal promyelocytes. An integrated approach using biochemical, radiological, flow cytometric, and molecular diagnosis drew our attention to plasma cell neoplasm. Thus, it is imperative to recognize these unusual morphologic characteristics to diagnose plasma cell neoplasms.

**Table 2:** Summary of previously described cases of plasma cells with azurophilic granules.

Author	Clinical profile	% of plasma cells	Immunophenotype	Diseases status
Nichols and Pozdnyakova^8^	66 y/male known case of IgG κ Multiple myeloma, post autologous stem cell transplant	13%	CD138, CD27, CD56, and κ immunoglobulin light chain restriction	Progression
Gupta et al^6^	68 y/male with bilateral lower limb paraesthesia and backache	20%	Polyclonal distribution for kappa and lambda	Reactive plasmacytosis
Shi and He^9^	74 y/female with fatigue	30%	CD138 and κ immunoglobulin light chain restriction	IgG κ-restricted multiple myeloma
Vlădăreanu et al^10^	80 y/male with back pain and progressive weakness	53%	CD38, CD138, CD56 positive	IgG κ-restricted multiple myeloma
Gralewski and Pina-Oviedo^2^	70 y/male with treated IgG κ plasma cell myeloma	12%	CD138 and κ immunoglobulin light chain restriction	IgG κ-restricted multiple myeloma
Ali and Moiz^11^	45 y/male with complaints of chest pain and weight loss	95%	MUM1 positive and negative for PAS and Sudan black B	IgG κ-restricted multiple myeloma
Sadeghi et al^12^	46 y/male progressive bone pain and night sweats	50%	CD138 positive	Plasma cell myeloma
Gajendra et al^13^	52 y/female with chest pain	3%	CD138 positive	IgG κ-restricted multiple myeloma
Juneja et al^3^	MGUS	NA	NA	NA

MGUS = monoclonal gammopathy of undetermined significance, MUM1 = multiple myeloma 1, PAS = periodic acid-schiff.
